# LRRK2-mediated mitochondrial dysfunction **in Parkinson’s disease**

**DOI:** 10.1042/BCJ20253062

**Published:** 2025-05-28

**Authors:** Silas A. Buck, Laurie H. Sanders

**Affiliations:** 1 Departments of Neurology and Pathology, Duke University School of Medicine, Durham, NC 27710, U.S.A; 2 Duke Center for Neurodegeneration and Neurotherapeutics, Duke University, Durham, NC 27710, U.S.A

**Keywords:** LRRK2, mitochondria, mitochondrial DNA, mitophagy, Parkinson’s disease

## Abstract

Parkinson’s disease (PD) is a neurodegenerative disorder characterized by motor symptoms including tremor, rigidity, and bradykinesia as well as degeneration of dopamine (DA) neurons in the substantia nigra *pars compacta* (SNc). A minority of PD cases are familial and are caused by a single genetic mutation. One of the most common PD-causing genes is leucine-rich repeat kinase 2 (*LRRK2*), which causes an autosomal dominant PD that presents very similarly to sporadic PD. Pathogenic mutations in *LRRK2* increase its kinase activity, indicated by both LRRK2 autophosphorylation and phosphorylation of its substrates. To date, the mechanism(s) by which elevated LRRK2 kinase activity induces DA neuron degeneration and PD has not been fully elucidated. One potential mechanism may involve the role of LRRK2 on mitochondria, as mitochondrial dysfunction has been linked to PD pathogenesis, and exciting recent evidence has connected PD pathogenic mutations in *LRRK2* to multiple aspects of mitochondrial dysfunction associated with the disease. In this review, we discuss the current knowledge implicating LRRK2 in mitochondrial energetics, oxidative stress, genome integrity, fission/fusion, mitophagy, and ion/protein transport in PD, as well as examine the potential role LRRK2 may play in mediating the effects of mitochondrial therapeutics being investigated for treatment of PD.

## Introduction

Parkinson’s disease (PD) is an age-associated neurodegenerative disorder affecting 0.5% of the population over 45 years old and 1–2% of the population over 65 years old [1,2], and its prevalence is rising rapidly as the population ages [3]. Clinically, PD is a movement disorder characterized by bradykinesia, rigidity, tremor, and gait disturbance, but non-motor symptoms are also common, including cognitive decline, depression, and pain [4]. These clinical symptoms are accompanied by 1) a loss of dopamine (DA) neurons in the substantia nigra *pars compacta* (SNc); 2) a loss of neurons in non-dopaminergic regions, like the pedunculopontine nucleus and locus coeruleus; and 3) accumulation of α-synuclein-containing protein aggregates, termed Lewy bodies [5,6]. Dysfunction and degeneration of projections from nigral DA neurons to the dorsal striatum contribute to PD motor symptoms [7–9], and DA precursors, like levodopa, are used to treat motor deficits resulting from this hypodopaminergic state [4]. While these medications may be effective at alleviating motor symptoms in PD, researchers have yet to discover a treatment that either halts or reverses PD neurodegeneration. Uncovering the molecular mechanisms underlying neurodegeneration in PD could lead to the development of effective treatments addressing the biological causes of PD.

Research of mechanisms underlying PD vulnerability has implicated many cellular processes in the disease, including proteostasis, lysosome, vesicular trafficking, and immune pathways [5,10,11]. Importantly, mitochondrial dysfunction has been identified as a key driver of PD pathogenesis [12–14]. Mitochondria regulate cellular energy production, reactive oxygen species (ROS) formation, calcium homeostasis, and cell death, among other functions [12–14]. The connection between mitochondrial dysfunction and PD first originated from the discovery that the heroin drug MPPP induces levodopa-responsive Parkinsonism in humans [15]. Subsequent work discovered that a by-product of MPPP, 1-methyl-4-phenyl-1,2,3,6-tetrahydropyridine (MPTP), is converted to MPP^+^ by astrocytes and transported into DA neurons through the DA transporter, where it inhibits mitochondrial complex I of the electron transport chain and induces DA neuron degeneration [16–20]. This mitochondria–PD connection has been substantiated by epidemiological evidence suggesting exposure to the pesticide rotenone, a potent mitochondrial complex I inhibitor, increases risk for developing PD [21]. Animal models of rotenone exposure have confirmed that rotenone induces DA neuron degeneration through mitochondrial complex I inhibition and subsequent ROS accumulation, as well as recapitulating α-synuclein accumulation, neuroinflammatory deficits, and autophagy-lysosomal dysfunction observed in human PD [22–24]. Strong epidemiological evidence has also been demonstrated for the pesticide paraquat increasing PD risk [21,24,25]. Like MPP^+^ and rotenone, animal models of paraquat exposure demonstrate that paraquat induces DA neuron degeneration [26]. However, paraquat differs from MPP^+^ and rotenone in that it induces mitochondrial stress from redox cycling [24,27,28]. The industrial solvent trichloroethylene (TCE) has recently been associated with PD risk [29,30], and while the mechanisms of action are less elucidated, in animal models, TCE is a mitochondrial toxicant that induces DA neuron degeneration [31,32]. Finally, other environmental toxicants are emerging both in their association with PD risk and in their impact on mitochondrial function [33]. Together, these epidemiological and animal model studies strongly implicate mitochondrial stress in PD risk.

While the majority of PD cases are idiopathic (iPD), approximately 10–20% of PD cases are familial [34–36]. Also, 2–5% of PD cases have a known monogenic cause [36,37], most commonly due to mutations in *LRRK2, SNCA, PRKN, PINK1, DJ-1, ATP13A2*, or *VPS35*. Additional genes confer increased risk for developing PD, especially *GBA1* [5,37]. Mutations in *PINK1* and *PRKN* disrupt mitophagy (removal and recycling of damaged mitochondria) as well as mitochondrial fission and fusion, while mutations in *DJ-1* affect mitochondrial function and mitochondrial oxidative stress [12,13]. In addition, the triplication and pathogenic mutations in *SNCA,* which encodes α-synuclein, affect mitochondria, and pathological α-synuclein aggregation leads to decreased mitochondrial ATP production, increased ROS, impaired mitochondrial protein import, and impaired mitochondrial calcium exchange [13,38–40], although it is unresolved whether α-synuclein is imported into the mitochondria to exert these effects [40,41]. Finally, the PD pathogenic *VPS35* mutation D620N impairs mitochondrial respiration, mitochondrial dynamics (fission/fusion), and mitophagy, and loss of *ATP13A2* increases mitochondrial fragmentation and ROS [42–47]. The mitochondrial role of many PD pathogenic mutants further demonstrates the importance of mitochondrial dysfunction in PD pathogenesis.

Compared with *PINK1, PRKN, DJ-1,* and *SNCA*, PD pathogenic mutations in *LRRK2* are more common, with the most common *LRRK2* mutation, Gly2019Ser (G2019S), accounting for 4% of familial and 1% of sporadic PD cases, and up to 36% of familial and 39% of iPD cases depending on ethnic background [10,48]. *LRRK2* mutations cause late-onset, autosomal dominant PD that is clinically and pathologically similar to iPD [49–51]. The *LRRK2* gene encodes leucine-rich repeat kinase 2 (LRRK2), a large multidomain protein with both kinase and GTPase activity. In human tissues, LRRK2 expression is highest in the lungs and kidneys [52]. At the cellular level, LRRK2 expression is high in peripheral blood mononuclear cells and neutrophils [53,54]. In the brain, LRRK2 expression is variable within neurons, with higher expression observed in striatal projection neurons and lower expression observed in striatal cholinergic and GABAergic interneurons, as well as ventral tegmental area (VTA) and SNc DA neurons [55–57]. LRRK2 is also strongly expressed in non-neuronal brain cells, including astrocytes and microglia [58]. At the sub-cellular distribution level, LRRK2 is most concentrated at membranous and vesicular structures, including synaptic vesicle-enriched and synaptosomal cytosolic fractions as well as lysosomes, endosomes, and, importantly, the mitochondrial outer membrane [59,60]. Considering that pathogenic *LRRK2* variants increase LRRK2 kinase activity, much research and clinical trials targeting LRRK2 have focused on reducing LRRK2 kinase activity and/or LRRK2 levels to treat both LRRK2 PD and iPD [10,61,62]. LRRK2 phosphorylates Rab proteins, and LRRK2 and its Rab substrates have been found to play roles in autophagy/lysosome function, vesicular trafficking, neurotransmission, centrosome cohesion and ciliogenesis, and immune responses [10,63]. Like other common genes associated with PD described above, recent work has uncovered an important role of LRRK2 in many mitochondrial processes. In this review, we discuss the current knowledge surrounding LRRK2’s role in mitochondria and the impact of pathogenic *LRRK2* mutations on mitochondrial dysfunction associated with PD pathogenesis.

### LRRK2 and mitochondrial energetics

The majority of neuronal ATP is produced via oxidative phosphorylation by the mitochondrial electron transport chain. SNc DA neurons, which are particularly vulnerable to PD-associated neurodegeneration, have higher baseline oxidative phosphorylation and ATP production than more resilient VTA DA neurons [64]. Pathogenic *LRRK2* mutations may contribute to DA neuron vulnerability through their effect on mitochondrial energetics, as primary DA neurons from PD pathogenic LRRK2 R1441G knock-in (KI) mice display a greater decrease in ATP levels in response to rotenone exposure than wildtype DA neurons [65]. Further, in rat primary midbrain neurons, of which ∼5% are DAergic, overexpression of G2019S LRRK2-GFP impairs both basal and ATP-linked oxygen consumption rate, indicating mitochondrial respiration impairment compared with the overexpression of GFP alone [66]. Additionally, striatal synaptosomes from LRRK2 R1441G KI mice have decreased cytochrome c oxidase subunit IV (COXIV) protein levels compared with wildtype mice, suggesting a mitochondrial energetic deficit due to decreased mitochondria levels in mutant LRRK2 DAergic projections *in vivo* [65]. Similar findings have been observed in non-DAergic neurons and non-neuronal brain cells as well. Primary cortical neurons from PD pathogenic LRRK2 R1441G KI mice display a greater rotenone-induced decrease in ATP levels [65], and neuroblastoma cells harboring the pathogenic G2019S LRRK2 mutation have decreased cellular ATP levels due to mitochondrial uncoupling [67]. Further, induced pluripotent stem cell (iPSC)-derived astrocytes from G2019S LRRK2 PD patients display decreased mitochondrial activity and ATP production [68]. Finally, even outside the brain, both pathogenic mutant *LRRK2* mouse- and human PD patient-derived fibroblasts display impaired mitochondrial energetics, evidenced by decreased ATP and mitochondrial membrane potential and impaired mitochondrial Complex I and Complex IV function [69–72]. Further, many of these deficits are not observed in non-manifesting *LRRK2* mutation carriers, suggesting LRRK2’s effect on mitochondrial energetics contributes to PD development [72]. Finally, *SNCA* triplication leads to mitochondrial respiration deficits, and LRRK2 may mediate pathological α-synuclein accumulation [13,38]. In all, these findings suggest pathogenic mutations in *LRRK2* impair mitochondrial energetics, regardless of cell type.

### LRRK2 and mitochondrial oxidative stress

ATP production via oxidative phosphorylation by the mitochondrial electron transport chain produces ROS in neurons [73]. This mitochondrial ROS production is critically important for healthy cellular processes and signaling [74]. However, excess ROS (or insufficient antioxidant activity) can cause oxidative stress, which is an imbalance between ROS accumulation and removal, and can damage cells through many mechanisms [75]. As described above, mitochondrial oxidative stress is implicated in PD, and SNc DA neurons have higher baseline mitochondrial ROS levels than resilient VTA DA neurons [64]. Increasing evidence suggests LRRK2 may be contributing to mitochondrial oxidative stress vulnerability of DA neurons. iPSC-derived DA neurons with the LRRK2 G2019S pathogenic mutation are more sensitive to oxidative stressors, including hydrogen peroxide (H_2_O_2_) and the dopaminergic neurotoxin 6-hydroxydopamine [76]. *In vivo*, TCE, an environmental mitochondrial toxicant linked to PD [31], induces oxidative stress (measured using dihydroethidium dye) and neurodegeneration in rat DA neurons, both of which are reversed by LRRK2 kinase inhibition [32]. Similarly, LRRK2 kinase inhibition in rats prevents lipid peroxidation, superoxide-generating NADPH oxidase 2 (NOX2) activation, and DA neuron degeneration in the rotenone PD model [77]. Together, these findings suggest that elevated LRRK2 kinase activity observed in LRRK2 PD increases DA neuron sensitivity to oxidative stressors, including those that target mitochondria like TCE and rotenone.

**Figure 1:**
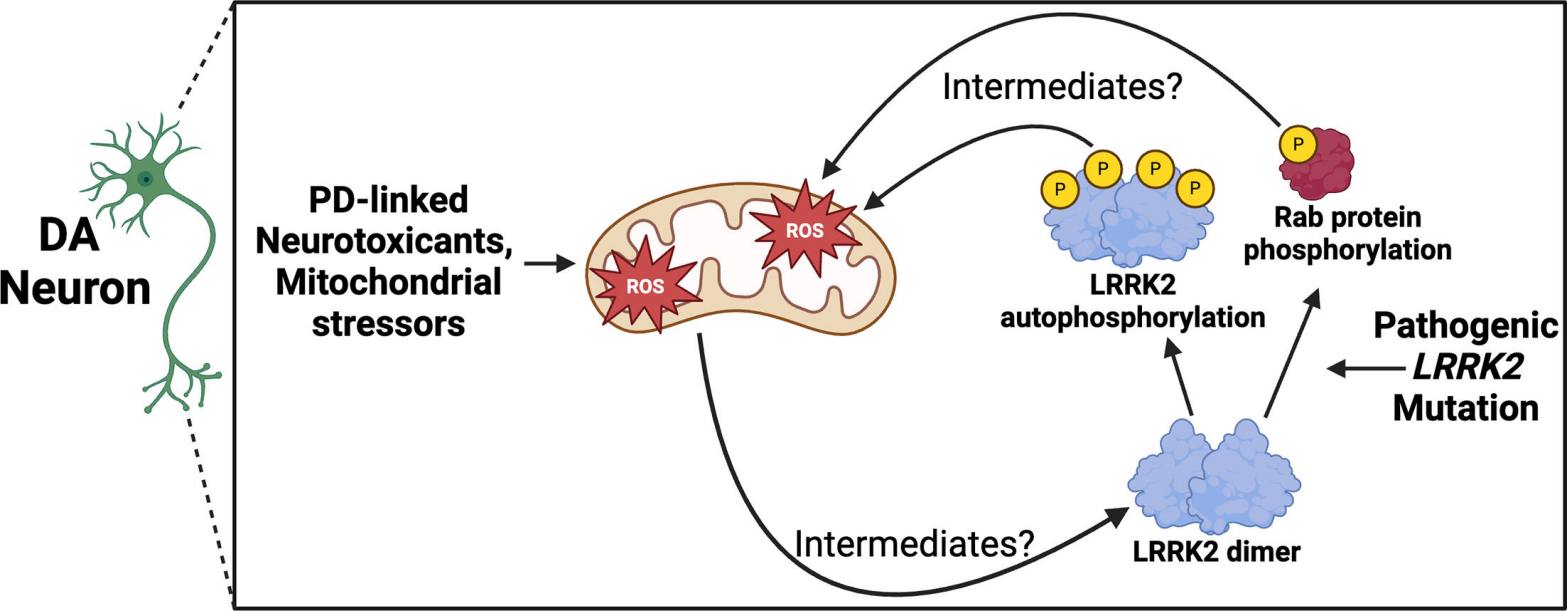
Positive feedback loop between mitochondrial ROS and LRRK2 kinase activation in DA neurons in PD. PD neurotoxicants induce mitochondrial ROS accumulation, which activates LRRK2 kinase activity via an undescribed mechanism (predominantly occurring when LRRK2 is a dimer [82]), leading to LRRK2 autophosphorylation and phosphorylation of Rab protein substrates. PD pathogenic *LRRK2* mutations also increase LRRK2 kinase activity. Increased LRRK2 kinase activity in turn increases mitochondrial ROS accumulation, also via an undescribed mechanism. DA, dopamine; LRRK2, leucine-rich repeat kinase 2; PD, Parkinson’s disease; PINK1, PTEN induced kinase 1; ROS, reactive oxygen species.

In parallel with these findings, similar work also suggests the reverse directionality, that is, oxidative stressors activate LRRK2 kinase activity. Both TCE and rotenone increase LRRK2 kinase activity in DA neurons, and this activation specifically derives from ROS [31,78]. Importantly, this TCE-induced increase in LRRK2 kinase activity (both LRRK2 autophosphorylation and Rab10 phosphorylation) occurs prior to DA neuron degeneration [31]. Consistent with this hypothesis, LRRK2 kinase activity is also increased by H_2_O_2_ and reduced by the antioxidant curcumin [78,79]. However, a recent paper identified two cysteine residues in LRRK2 that regulate its redox sensitivity, and these residues mediate a down-regulation of LRRK2 kinase activity in response to oxidizing agents like H_2_O_2_ [80]. In either case, these findings have led to the proposal that LRRK2 is a reduction/oxidation (redox)-sensitive protein [81]. LRRK2 may therefore contribute to DA neuron degeneration in PD via a positive feedback loop, wherein mitochondrial stress increases ROS, increasing LRRK2 kinase activity, which further increases vulnerability to mitochondrial oxidative stress (Figure 1). Further, mitochondrial stress may contribute to lysosomotropic agent-induced LRRK2 localization to lysosomes, although this has not been tested.

Outside of DA neurons, an effect of LRRK2 on mitochondrial oxidative stress has been observed in human samples as well as in lower organisms and *in vitro* models. An increase in oxidative stress markers, namely 8-hydroxy-2′-deoxyguanosine (8-OHdG) and 8-isoprostane (8-ISO), has been observed in cerebrospinal fluid from healthy participants that harbor the *LRRK2* G2019S mutation (i.e., non-manifesting carriers) [83]. In human dermal fibroblasts, expression of the transcription factor nuclear factor erythroid 2-related factor 2 (Nrf2), which regulates antioxidant signaling, is increased in LRRK2 G2019S PD patients, but not non-PD-manifesting carriers, compared with control participants [84], suggesting mitochondrial oxidative stress vulnerability may be a mechanism underlying LRRK2 PD penetrance, at least with regard to the G2019S mutation. In yeast, overexpression of human LRRK2 is associated with increased resistance to H_2_O_2_, which is attenuated in yeast overexpressing human LRRK2 G2019S or R1441C pathogenic mutants [85]. In *Drosophila melanogaster*, glutamate-cysteine ligase (Gcl) expression boosts levels of the antioxidant glutathione, which is associated with lifespan in naturally varying *Drosophila* strains. Gcl levels are reduced in LRRK2 G2019S PD patient brains [86]. In nerve-like differentiated cells, the LRRK2 inhibitor PF-06447475 reverses rotenone-induced ROS accumulation [87]. Further, iPSCs from G2019S and R1441C LRRK2 PD patients display a higher oxidative stress susceptibility and reduced basal oxygen consumption rate that is reversed by LRRK2 inhibition [88], and neural stem cells from pathogenic R1441G LRRK2 mice display higher oxidative stress [89]. Wildtype LRRK2 overexpression also increases ROS in mouse primary cortical neurons, and G2019S LRRK2 overexpression increases ROS greater than wild-type LRRK2 [90]. Importantly, mitochondrial ROS is increased in R1441C LRRK2 rat primary cortical neurons [91]. Finally, LRRK2 mediates PD-linked toxicant-induced ROS accumulation and lipid peroxidation in HEK293 cells, and LRRK2 expression restores toxicant-induced ROS production in LRRK2 KO HEK293 cells [32,77]. More specifically, LRRK2 kinase activity both promotes mitochondrial ROS production and activates NADPH oxidase 2 (NOX2), which is the major source of ROS accumulation in LRRK2 G2019S HEK293 cells [77].

LRRK2 may contribute to mitochondrial oxidative stress vulnerability in the PD brain via its impact on non-neuronal cells as well, especially macrophages/microglia. LRRK2 inhibition by MLi-2 reduces lipopolysaccharide-induced microglial activation and subsequent neuronal ROS production [92]. The pathogenic G2019S *LRRK2* mutation elevates ROS in macrophages [93], and in aged female mice, the pathogenic *LRRK2* R1441C mutation induces differential expression of mitochondrial and oxidative stress genes in macrophages compared with wildtype macrophages [94]. Conversely, LRRK2 knockout (KO) macrophages are vulnerable to mitochondrial oxidative stress due to reduced antioxidant pools [95]. In addition, an exciting recent paper proposes a novel mechanism whereby microglia use tunneling nanotubes to connect with neurons burdened by pathological protein aggregates like α-synuclein, enabling donation of healthy mitochondria to restore mitochondrial function and reverse ROS elevation and associated mitochondrial oxidative stress [96]. Importantly, this mitochondrial rescue is compromised in G2019S LRRK2 mutant microglia [96]. Finally, LRRK2 may contribute to astrocytic mitochondrial oxidative stress as well, as iPSC-derived astrocytes from G2019S LRRK2 PD patients display aberrant mitochondrial morphology and increased ROS production [68].

### LRRK2 and mitochondrial genome integrity

Many nuclear-encoded genes localize to the mitochondria, and variation in these genes is known to affect the age of onset of LRRK2 PD [97]. In addition to the nuclear genome, mitochondria contain their own double-stranded, circular DNA (mitochondrial DNA or mtDNA) comprising 37 genes, which is transmitted through the female germline [98]. DNA damage accumulation and dysfunctional DNA repair are observed in PD [99]. This includes evidence of mtDNA damage, in the form of mtDNA 8-oxo-deoxyguanine or abasic sites, and mitochondrial mutagenesis (i.e., deletions, point mutations, and transversions) in SNc DA neurons of PD patients [100–105]. Relatedly, neuronal mtDNA damage is observed in the context of PD-linked environmental toxicants paraquat [106] and rotenone [100,107], as well as 6-hydroxydopamine [108], MPTP [109], α-synuclein pathology [110], or cellular DA oxidation [111,112].

Over the last decade, a strong line of evidence has implicated PD pathogenic LRRK2 in inducing mtDNA damage and/or mutations. mtDNA damage is increased in G2019S LRRK2 PD patient iPSC-derived neural cells, lymphoblastoid cells, peripheral blood mononuclear cells, and HEK293 cells [113–115]. Rat primary midbrain neurons (but not cortical neurons) overexpressing G2019S LRRK2 also increase mtDNA damage, but importantly, this elevation of mtDNA damage is also observed under endogenous conditions [66,113–115]. Increased LRRK2 G2019-dependent mtDNA damage is reversed by gene correction, LRRK2 kinase inhibition (even at low concentrations), and LRRK2 G2019S-selective kinase inhibition [66,113–115]. mtDNA damage can (but does not always) convert to a mutation [116]. Nevertheless, mtDNA mutations are observed in fibroblasts from G2019S LRRK2 carriers, with higher levels observed in PD-manifesting carriers than non-manifesting carriers [117]. Additionally, both *LRRK2* mutation carriers and iPD patients exhibit alterations in mtDNA transcription and replication, further demonstrating a similarity between iPD- and LRRK2 PD-associated mtDNA dysfunction [118]. These findings suggest LRRK2’s role in mtDNA damage in both iPD and LRRK2 PD, but to date, research comparing various facets of mitochondrial dysfunction in LRRK2 PD-manifesting and non-manifesting carriers is limited. Future work should explore the similarities and differences between PD-manifesting and non-manifesting carriers to reveal whether aspects of mitochondrial genome integrity and dysfunction are a prerequisite for conversion to PD.

The sensitivity of mtDNA damage changes in response to modulation of LRRK2 kinase activity suggested mtDNA damage could serve as a biomarker of LRRK2 PD and potentially iPD as well. To test this hypothesis, we developed a PCR-based mtDNA damage assay (MitoDNA_Dx_) to accurately quantify mtDNA damage in a scalable platform [107]. Using this assay, we found that both G2019S LRRK2 PD patient- and iPD patient-derived peripheral blood mononuclear cells displayed increased mtDNA damage compared with controls, including in iPD patients with acute washout of PD-related medications. Notably, LRRK2 kinase inhibition reversed mtDNA damage in iPD patient-derived lymphoblastoid cells, suggesting LRRK2 kinase activity contributes to mtDNA damage in PD patients without a pathogenic *LRRK2* mutation [107]. Consistent with previous findings of elevated mtDNA mutations in G2019S LRRK2 non-PD-manifesting carriers [117], we observed an increase in mtDNA in G2019S LRRK2 non-manifesting carriers as well, establishing an increase in mtDNA damage is associated with pathogenic LRRK2, irrespective of PD diagnosis. Similar findings were observed in G2019S LRRK2 KI mouse ventral midbrain (a region containing the SNc) compared with wildtype, along with decreased mtDNA damage in LRRK2 KO mouse ventral midbrain compared with wildtype [107]. No mtDNA damage was observed in patients with Alzheimer’s disease, suggesting mtDNA damage accumulation is specific to PD-associated neurodegenerative processes. Finally, mtDNA damage was stable and consistent within and between individuals over time, demonstrating mtDNA damage is relatively stable in the absence of neurodegenerative disease [107]. In all, these findings highlight mtDNA damage as 1) a shared blood-based biomarker of iPD and LRRK2 PD, and 2) a potential blood-based biomarker of LRRK2 inhibition pharmacodynamics. These discoveries also highlight the importance of mitochondria in PD and in mediating the role of LRRK2 and LRRK2 kinase activity in both LRRK2 PD and iPD.

The mechanism by which LRRK2 exerts its impact on mtDNA damage and/or repair has not been elucidated. One possibility is that LRRK2’s impact on the outer mitochondrial membrane and role in mitochondrial dynamics and mitophagy may indirectly lead to mtDNA damage accumulation. Alternatively, LRRK2 has been shown to interact with the autophagy receptor p62/SQSTM1 *in vitro* [119], and p62/SQSTM1 was found to suppress DNA double-strand break repair when dysregulated [120]. It is important for future work to uncover the mechanism(s) by which LRRK2 contributes to mtDNA damage accumulation in PD, as targeting the LRRK2-mitochondria-mtDNA connection could lead to treatments for addressing LRRK2 and mitochondrial dysfunction, and subsequent DA neuron degeneration, in both LRRK2 PD and iPD.

Important lessons, specifically regarding the impact of mtDNA dysfunction on PD pathogenesis, can be learned from inherited mitochondrial diseases. For example, Leigh syndrome is the most common mitochondrial disorder affecting children and can result from mutations in multiple different nuclear and mitochondrial genes which induce deficits in mitochondrial oxidative phosphorylation through affecting the electron transport chain, including complex I [121]. The nigrostriatal DA pathway has been implicated in Leigh syndrome pathogenesis, as 1) some Leigh syndrome patients display motor symptoms, including dystonia and ataxia [122–126], 2) characteristic brain imaging changes in Leigh syndrome include abnormalities and atrophy in the striatum [121,126], 3) acute exposure to the PD toxicant MPTP has been explored as a model for Leigh syndrome [127], and 4) the PD drug and DA agonist apomorphine protects against ROS-associated cell death in Leigh syndrome patient-derived fibroblasts [128]. However, there are important differences between mitochondrial diseases and LRRK2-associated mtDNA dysfunction. For example, in MELAS (mitochondrial encephalomyopathy, lactic acidosis and stroke-like episodes) syndrome, Leber’s Hereditary Optic Neuropathy (LHON), Leigh syndrome, and many other genetic mitochondrial diseases, like mitochondrial DNA depletion syndromes, alterations in mtDNA copy number and heteroplasmy in mitochondrial mutations are observed and are linked to disease penetrance, clinical outcomes, and severity of disease [129–133]. However, the directionality of changes in mtDNA copy number and heteroplasmy is not as clear and has been shown to be decreased, not changed, or even increased in LRRK2 PD [84,107,117,134–136]. The magnitude of mutated mtDNA copies and absolute copy number also differs between inherited primary mitochondrial disorders and LRRK2 PD. Importantly, due to mtDNA heteroplasmy and the potential cell type and tissue specificity of mitochondrial phenotypes, it is critical for future studies to consider the source of the sample, preparation, and technical and methodological differences of assays to resolve the variation in findings in LRRK2 PD and mitochondrial diseases [132].

### LRRK2 and mitochondrial fission/fusion

Mitochondria routinely undergo fission and fusion to maintain proper mitochondrial function, and disruption in this balance between fission and fusion can contribute to mitochondrial dysfunction. PD pathogenic *LRRK2* mutations alter mitochondrial fission and fusion, but results are contradictory. Some studies find that the G2019S *LRRK2* mutation induces mitochondria elongation in human PD fibroblasts and aged mouse striatum [70,137]. Decreased fission protein levels are observed in aged LRRK2 G2019S mouse brain as well [137]. Further, LRRK2 inhibition induces mitochondrial fission in SH-SY5Y cells [138].

**Figure 2:**
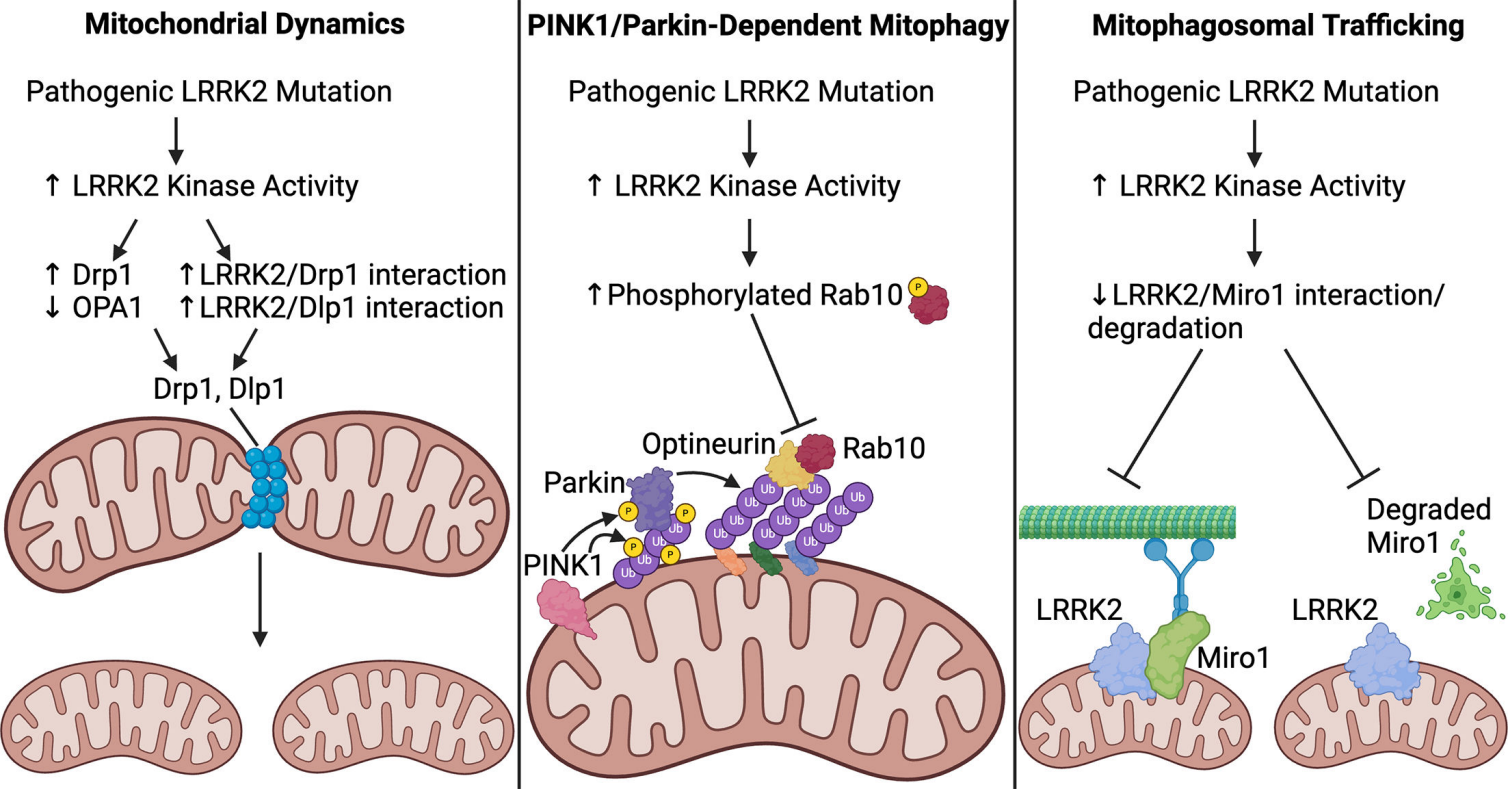
Impact of LRRK2 on mitochondrial dynamics and quality control. (**A**) Increased LRRK2 kinase activity from pathogenic *LRRK2* mutations increase Drp1 while decreasing OPA1 levels and increases LRRK2 interaction with Drp1 and Dlp1; these actions all promote mitochondrial fission. (**B**) Elevated LRRK2 activity increases Rab10 phosphorylation, which inhibits Rab10 interaction with optineurin, halting PINK1/Parkin-dependent mitophagy. (**C**) Pathogenic LRRK2 mutations decrease LRRK2 interaction with Miro1 and/or degradation of Miro1, impairing mitochondrial anchoring to microtubule motor proteins and preventing mitophagy initiation following Miro1 degradation. LRRK2, leucine-rich repeat kinase 2; PINK1, PTEN induced kinase 1.

On the other hand, wildtype LRRK2 overexpression in mouse primary cortical neurons induces mitochondrial fission, and this increase is exacerbated by the G2019S mutation [90]. iPD patient-derived fibroblasts display mitochondrial fragmentation that is reversed by LRRK2 inhibition [139], and mitochondrial connectivity is reduced in G2019S LRRK2 PD fibroblast cultures [140]. Also in contrast with the above findings, SH-SY5Y cells expressing LRRK2 G2019S or R1441C exhibit mitochondrial fragmentation with shorter and smaller mitochondria [141], and similar decreases in mitochondria size are observed in R1441C LRRK2 mouse SNc [142] and R1441G LRRK2 mouse striatum [143]. Ectopic expression of G2019S LRRK2 in BV2 cells fragments mitochondria and increases expression of Drp1, a mitochondrial fission protein [144]. Relatedly, LRRK2 KO in HEK293 cells reduces Drp1 localization with mitochondria [145]. The novel LRRK2 variant E193K, identified in an Italian family with PD, increases LRRK2 binding to Drp1 [146], although this mutation induces decreased LRRK2 binding to Drp1 in response to MPP^+^ exposure, impairing mitochondrial fission. In addition to Drp1, LRRK2 interacts with Dlp1 to induce mitochondrial fragmentation [90,141], and levels of OPA1, a mitochondrial fusion protein, are reduced in brains of LRRK2 G2019S PD patients [147]. Finally, LRRK2 may induce mitochondrial fission through its substrate Rab7, as Rab7 GTP hydrolysis regulates mitochondrial fission at mitochondria-lysosome contacts [148]. In all, while conflicting findings have not been resolved, the majority of evidence points toward an imbalance towards mitochondrial fission in pathogenic mutant LRRK2 models and LRRK2 PD (Figure 2A).

### LRRK2 and mitochondrial quality control/mitophagy

Mitophagy refers to autophagy of mitochondria, that is, the delivery of damaged, dysfunctional, or unneeded mitochondria to the lysosome for degradation [149]. Mitophagy occurs both under basal conditions and in response to mitochondrial stress, via PTEN-induced kinase 1 (PINK1)/Parkin-dependent mitophagy as well as PINK1/Parkin-independent mitophagy. Mitophagy is heavily implicated in PD pathogenesis, as mutations in both *PINK1* and *Parkin* cause autosomal recessive, early-onset PD [37]. LRRK2’s role in mitophagy (reviewed in-depth in [150]) can be categorized by LRRK2’s impact on (1) basal mitophagy, (2) PINK1/Parkin-dependent mitophagy, and (3) mitophagosome trafficking and lysosomal fusion.

Recent evidence has found an association of LRRK2 with basal mitophagy. In LRRK2 G2019S mouse embryonic fibroblasts, brain, kidney, and lung, basal mitophagy is impaired in a kinase-dependent manner, independent of the PINK1 pathway [151,152]. Basal mitophagy is also decreased in LRRK2 R1441C rat primary cortical neurons and R1441C LRRK2 PD iPSC-derived DA neurons [91]. However, basal mitophagy was unchanged in R1441C iPSC-derived cortical neurons [91]. Importantly, both Type I LRRK2 kinase inhibitors (which bind to the kinase domain in a closed active conformation) and Type II LRRK2 kinase inhibitors (which maintain the kinase in an open inactive conformation) stimulate basal mitophagy in wildtype and G2019S LRRK2 KI mouse embryonic fibroblasts, but not LRRK2 KO fibroblasts [151,152]. These basal mitophagy deficits are also independent of changes in general macroautophagy, suggesting a selectivity for LRRK2 in mediating mitophagy [151,153]. Despite these positive findings, it should be noted that basal mitophagy was not altered in homozygous G2019S LRRK2 KI HEK293 cells, in G2019S LRRK2 PD patient fibroblasts, or in R1441C LRRK2 IPSC-derived DA neurons compared with controls [91,114,154]. More work is needed to address discrepancies in these findings to determine LRRK2’s impact on basal mitophagy.

In PINK1/Parkin-dependent mitophagy, PINK1 acts as a damage sensor and accumulates on the cytosolic side of the outer mitochondrial membrane, where it phosphorylates both ubiquitin at Serine 65 (pS65-ubiquitin) and then Parkin at Serine 65 (after it binds pS65-ubiquitin) [155–159]. Parkin then ubiquitylates multiple proteins on the outer mitochondrial membrane, recruiting autophagy machinery via ubiquitin-binding autophagy receptors like optineurin, resulting in the formation of the mitophagosome [149,160,161]. The mitophagosome is degraded after fusing with the lysosome, thus completing PINK1/Parkin-dependent mitophagy [162]. A substantial body of work supports a role of LRRK2 in PINK1/Parkin-dependent mitophagy (Figure 2B). G2019S LRRK2 impairs PINK1/Parkin-dependent mitophagy in COS7 cells and G2019S LRRK2 PD patient fibroblasts in a LRRK2 kinase- and Drp1-dependent manner [154,163]. LRRK2 KO and LRRK2 inhibition attenuate TCE-induced mitophagy deficits in HEK293 cells, and LRRK2 inhibition reduces the abundance of mitochondria labeled by PINK1/Parkin mitophagy marker pS65-ubiquitin in TCE-exposed rat SNc DA neurons [32]. Importantly, LRRK2-associated DA neuron degeneration may be caused by LRRK2’s effect on PINK1/Parkin-dependent mitophagy, as co-expression of human Parkin in LRRK2 G2019S transgenic *Drosophila* protects against DA neuron degeneration [164]. More generally, pathogenic *LRRK2* mutations affect mitochondrial depolarization-induced mitophagy, which includes, but is not limited to, PINK1/Parkin-dependent mitophagy. Homozygous G2019S LRRK2 KI HEK293 cells and G2019S LRRK2 PD patient fibroblasts display a deficit in mitochondrial depolarization-induced mitophagy [114,163]. Further, R1441G LRRK2 impairs mitochondrial depolarization-induced mitophagy in mouse embryonic fibroblasts, although this deficit was not reversed by LRRK2 kinase inhibition [143]. LRRK2 inhibition also increases mitochondrial depolarization-induced mitophagy in wildtype LRRK2 human fibroblasts [163].

While LRRK2 may mediate its effects on PINK1/Parkin-dependent mitophagy by directly interacting with Parkin [165], an intriguing study found that the LRRK2 substrate Rab10 accumulates on depolarized mitochondria in a PINK1- and Parkin-dependent manner, where it binds optineurin and promotes mitophagy [153]. Pathogenic LRRK2 mutations increase Rab10 phosphorylation, which impairs 1) Rab10 translocation to mitochondria, 2) Rab10 association with optineurin, and 3) PINK1/Parkin-dependent mitophagy [153]. This work presents a comprehensive mechanism by which pathogenic mutant LRRK2 impairs PINK1/Parkin-dependent mitophagy via Rab10. It is important to note that LRRK2 localizes to the outer mitochondrial membrane and therefore may play a role in mitophagy independent of Rab10 as well [60]. Future work could use mass spectrometry and related proteomics approaches to comprehensively investigate changes to outer mitochondrial membrane protein content, or even total mitochondrial protein content, in LRRK2 PD. This could uncover candidates that mediate LRRK2’s effect on mitophagy and other mechanisms of mitochondrial dysfunction in LRRK2 PD and iPD.

LRRK2 and its Rab substrates have been implicated in vesicle transport [166], including lysosomes [167,168] and autophagosomes [169,170], which probably affect mitophagy separate from a direct effect of LRRK2 at the mitochondria. Interestingly, the *LRRK2* G2019S mutation impairs mitochondrial trafficking in both the anterograde and retrograde directions in iPSC-derived G2019S LRRK2 PD neurons [171]. This is due to the G2019S mutation disrupting a complex that LRRK2 forms with Miro1, a mitochondrial outer membrane protein that, in addition to mediating calcium signaling, anchors mitochondria to microtubule motors as an early step in mitophagy [171]. Interestingly, a separate study observed that LRRK2 interaction with Miro1 was not perturbed in IPSC-derived DA neurons with *LRRK2* R1441C mutation. Despite the lack of change in LRRK2/Miro1 interaction, *LRRK2* R1441C mutation impaired Miro1 degradation [91]. Importantly, Miro1 degradation occurs after microtubule anchoring to mitochondria, and this degradation initiates mitophagy [91]. Together, these findings suggest that pathogenic LRRK2 impairs mitophagy through Miro1 (Figure 2C), although the nature of LRRK2’s effect on Miro1 may depend on the specific pathogenic mutation. It should be noted that another study found that iPSC-derived G2019S LRRK2 PD neurons display an increased percentage of mitochondria that are mobile compared with non-mutant neurons [88], although this is a different measurement than the transport speed of individual mitochondria.

### LRRK2 and mitochondrial calcium transport and protein import

In addition to oxidative phosphorylation, mitochondria play a key role in calcium (Ca^2+^) buffering [172,173]. SNc DA neurons vulnerable to PD have slow, rhythmic spiking accompanied by large oscillations in intracellular Ca^2+^ concentration, promoting Ca^2+^ entry into the mitochondria, which in turn stimulates oxidative phosphorylation and ATP production [6]. This process contributes to vulnerability to degeneration as elevated mitochondrial Ca^2+^ triggers pore opening and apoptosis [174], and Ca^2+^ accumulation directly promotes α-synuclein aggregation [175,176]. Primary mouse cortical neurons expressing pathogenic G2019S or R1441C *LRRK2* mutations up-regulated mitochondrial Ca^2+^ uptake proteins, which was coupled with increased mitochondrial Ca^2+^ uptake and neurite retraction [177]. These findings were replicated in LRRK2-mutated patient fibroblasts [177].

LRRK2 can also form a complex with Miro1 [171], and Miro1 is an outer mitochondrial membrane protein that regulates mitochondria-endoplasmic reticulum contact and associated calcium exchange and signaling [178–181]. The formation of this LRRK2-Miro1 complex is impaired with the *LRRK2* G2019S mutation [171], and Miro1 has been implicated in PD pathogenesis [182]. Notably, mutations in Miro1 found in PD patients decrease mitochondrial–endoplasmic reticulum contact and impair calcium homeostasis [183]. In addition to affecting mitochondrial calcium import, LRRK2 may also negatively affect mitochondrial protein import through either a direct or an indirect effect on α-synuclein. Pathological α-synuclein binds to translocase of the outer membrane 20 (TOM20), preventing TOM20’s interaction with TOM22 and impairing mitochondrial import of nuclear proteins important for proper mitochondrial function, like Ndufs3 [40,184]. Thus, pathogenic LRRK2 may impair mitochondrial protein import by increasing α-synuclein aggregation, which could be tested via proteomic study of mitochondria in LRRK2 models and analyzing changes in mitochondrial protein content.

LRRK2 and α-synuclein have multiple common roles in mitochondrial function that may explain mitochondrial deficits observed in LRRK2 PD. These roles include mitochondrial protein import, mitochondrial energetics, mitochondrial oxidative stress vulnerability, mtDNA damage, and mitochondrial calcium dynamics [38–40,66,107,110,113–115]. Further, LRRK2 may directly affect α-synuclein misfolding and accumulation, as mice and rats with pathogenic *LRRK2* mutations display increased accumulation of insoluble α-synuclein inclusions and α-synuclein-induced neurodegeneration, which can be reversed by LRRK2 kinase inhibition [185–187]. On the other hand, some studies show that α-synuclein inclusion formation is independent of pathogenic LRRK2 [188,189]. In human LRRK2 PD, α-synuclein pathology is commonly observed, although variable [190–192]. However, some studies have suggested that early (pre-Lewy body) α-synuclein aggregates, specifically α-synuclein oligomers, contribute to neurodegeneration [193–195], and small soluble aggregates of α-synuclein have been isolated from a LRRK2 PD patient without Lewy bodies [196]. Indeed, through a proximity ligation assay for α-synuclein, recent work has shown substantial α-synuclein pathology in brains of LRRK2 PD patients that are negative for Lewy bodies [197,198]. However, considering approximately one third of LRRK2 PD patients are negative on the α-synuclein seed amplification assay derived from cerebrospinal fluid [199], future work should reconcile these differences. It would also be interesting to investigate whether positive vs. negative α-synuclein seed amplification assay results explain the distribution in mtDNA damage levels observed in LRRK2 PD and non-manifesting *LRRK2* mutation carriers [107]. Finally, tau is also a commonly observed co-pathology in LRRK2 PD [190], and tau aggregation induces mitochondrial deficits as well [200–202]. Therefore, future studies are required to disentangle the distinct roles of LRRK2, α-synuclein, and tau in mediating mitochondrial dysfunction in LRRK2 PD.

### Role of LRRK2 in PD therapeutics targeting mitochondria

Based on this strong connection between PD and mitochondrial dysfunction, clinical trials have sought to address mitochondrial dysfunction to treat PD. To date, however, all such treatments have failed, although recent attempts show some promise (reviewed in [13]). Most therapeutics have centered on mitochondria-associated oxidative stress and mitochondrial quality control. Considering LRRK2’s role in both, we will review the potential impact of pathogenic LRRK2 on these mitochondrial therapies as well as discuss the mitochondrial impact of LRRK2 inhibitors that are currently in clinical trials.

Many antioxidant approaches have been tested as potential treatments of PD, including coenzyme Q_10_, MK-7, EPI-589, and MitoQ [203–208]. As described above, the pathogenic *LRRK2* mutation increases oxidative stress vulnerability, so PD patients with a *LRRK2* mutation or iPD subjects with higher LRRK2 kinase activity may be more likely to positively respond to these treatments, taking a precision medicine-based approach. While not yet in the clinical trial phase, multiple potential therapeutics targeting mitophagy have direct connections to LRRK2. First, mitochondrial division inhibitor 1 (mdivi-1) blocks the mitochondrial fission protein Drp1, leading to reduction of mitochondrial fragmentation, reduction of mitochondrial ROS, increased ATP production, and attenuated DA neuron degeneration in *in vitro* and *in vivo* α-synuclein, rotenone, and MPP^+^ PD models [209–212]. Importantly, mdivi-1 also induces DA neuroprotection in a PINK1 KO mouse model [212]. Mdivi-1 may be more efficacious for PD patients with *LRRK2* mutations or increased LRRK2 kinase activity, as LRRK2 binds to Drp1 to promote mitochondrial fission, and this effect is exacerbated with the LRRK2 G2019S mutat ion [144–146]. Second, reduction of Miro1, either through RNA interference or small molecule binding, rescues mitophagy deficits and degeneration in *Drosophila* and human α-synuclein PD models and human PD fibroblasts [213,214]. Like Drp1, LRRK2 interacts with Miro1, and this interaction is disrupted by the LRRK2 G2019S mutation [171,178–181]. Finally, studies increasing PINK1 or Parkin levels have observed rescue from MPP^+^-associated neurodegeneration and α-synuclein-associated toxicity [215–219], and LRRK2 may modulate these effects through its role in PINK1/Parkin-dependent mitophagy [150]. In terms of therapies currently in the clinical trial pipeline, multiple treatments targeting mitochondria are in Phase 1 or 2 clinical trials, including PINK1 inhibitors (NCT06414798), intrastriatal implantation of mitochondrial health-promoting stem cells (NCT05094011), glutathione (and insulin), nicotinamide riboside, and terazosin [220]. Additionally, multiple mitochondrial-targeted therapies relevant to PD are currently in pre-clinical development.

**Figure 3:**
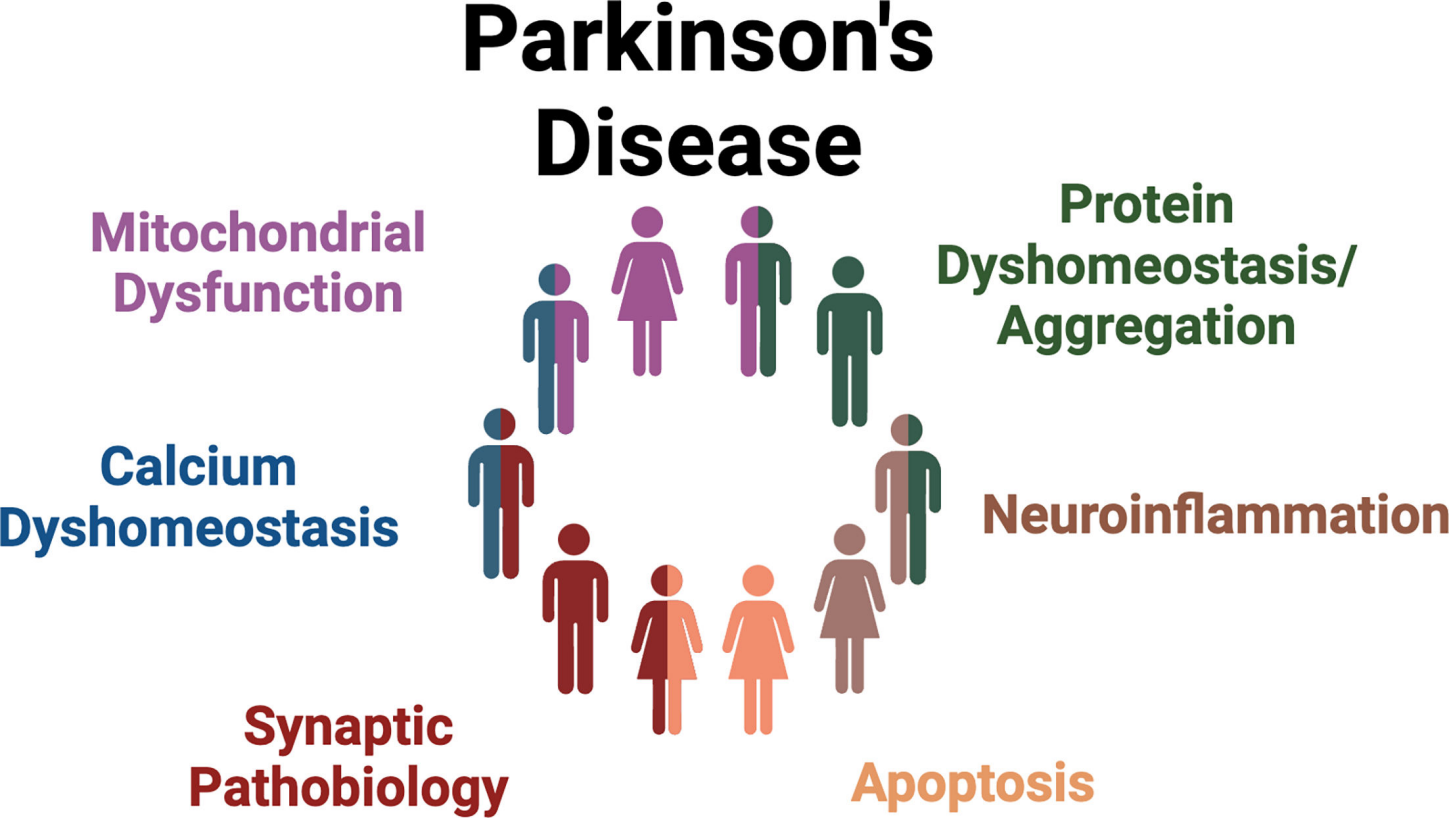
PD stratification in clinical trial development. PD pathogenesis likely stems from early pathogenic molecular abnormalities that differ between patients [222]. Future work should aim to stratify PD patients based on these different underlying abnormalities. PD, Parkinson’s disease.

LRRK2 inhibitors, antisense oligonucleotides, and proteolysis targeting chimeras (PROTACs) are currently in various stages of clinical development [61,221]. Considering the strong role of LRRK2 in PD-associated mitochondrial dysfunction reviewed here, it may be beneficial to analyze mitochondrial dysfunction as a biomarker of both LRRK2 target engagement and/or therapeutic efficacy. For example, mtDNA damage levels in peripheral blood mononuclear cells derived from PD patients can be analyzed via the MitoDNA_Dx_ assay and used as a biomarker of LRRK2 kinase activity [107]. LRRK2’s central role in mitochondrial dysfunction associated with PD, coupled with the previous failures in developing mitochondrial therapeutics for PD, highlights the necessity to acknowledge heterogeneity in PD pathogenesis and develop treatments that target PD stratification based on different biological sources of PD etiology (Figure 3) [222–225]. The development of reliable biomarkers like the α-synuclein seed amplification assay, LRRK2 kinase activity biomarkers, and the MitoDNA_Dx_ assay will be essential in patient stratification. If these patient stratification and biomarker strategies are successfully developed, LRRK2 PD therapeutics could be used alone or in combination to treat each patient individually (i.e., through precision medicine). For example, in the context of LRRK2 and mitochondria, mitochondrial treatments may be more likely to succeed in iPD patients with elevated LRRK2 kinase activity, and conversely, LRRK2 kinase inhibitors may be more likely to succeed in iPD patients with increased mtDNA damage as assessed by the MitoDNA_Dx_ assay. A more biologically- and stratification-driven treatment development strategy like this could provide increased success in clinical trials and one day lead to a successful precision medicine approach in PD.

## Conclusions

Over the past decade, much progress has been made in uncovering both mitochondrial disturbances underlying PD pathogenesis as well as the role of LRRK2 in PD-associated mitochondrial dysfunction. However, outstanding questions remain, particularly regarding the mechanism(s) by which 1) pathogenic LRRK2 induces mitochondrial vulnerability, 2) mitochondrial stress activates LRRK2 kinase activity, and 3) mitochondrial stress leads to selective neurodegeneration of SNc DA neurons. Additionally, future work should investigate the potential for stratification of PD participants by biological classifications regarding mitochondrial dysfunction, ubiquitin-proteasome dysfunction, neuroinflammation, and so on. Different biological processes may drive PD pathogenesis in different patients, so stratification is needed to create more targeted and more effective treatment approaches. In all, *LRRK2* mutations associated with PD are fundamentally linked to mitochondrial dysfunction and subsequent neurodegeneration, opening an avenue for investigating LRRK2–mitochondria interactions for the purpose of targeting PD patients with elevated LRRK2 kinase activity and/or deficits in mitochondrial function.

## Abbreviations

DA: dopamine
H_2_O_2_: hydrogen peroxide
KI: knock-in
KO: knockout
LRRK2: leucine-rich repeat kinase 2
PD: Parkinson’s disease
PINK1: PTEN induced kinase 1
ROS: reactive oxygen species
SNc: substantia nigra pars compacta
TCE: trichloroethylene
iPD: idiopathic Parkinson’s disease
iPSC: induced pluripotent stem cell
mdivi-1: mitochondrial division inhibitor 1
mtDNA: mitochondrial DNA
